# Research on the Influence of Different Wax-Based Warm Mix Additives on Rheological and Aging Behaviors of High-Viscosity Modified Asphalt

**DOI:** 10.3390/polym18050646

**Published:** 2026-03-06

**Authors:** Jingqing Huang, Bei Chen, Yingchun Cai, Jinchao Yue, Bishuai Hong, Guoqi Tang

**Affiliations:** 1 School of Water Conservancy and Transportation, Zhengzhou University, Zhengzhou 450001, China; huangjq200811@126.com (J.H.); yccai@zzu.edu.cn (Y.C.); yuejc@zzu.edu.cn (J.Y.); bshong1@163.com (B.H.); 2 Guolu Gaoke Engineering Technology Institute Co., Ltd., Beijing 100083, China; tang12341102@163.com

**Keywords:** high-viscosity modified asphalt, warm mix agent, aging performance, rheological properties, warm mix effect

## Abstract

This study introduces five types of wax materials to replace traditional Sasobit warm mix agents (WMAs), aiming to reduce the aging performance of high-viscosity modified asphalt (HMA) under high temperatures and optimize wax-based WMAs for a better warm mix effect and more stable performance of HMA. In this study, styrene–butadiene–styrene (SBS) modifier was first used to prepare HMA, and then wax materials were added to prepare HMA. Thin-Film Oven Tests (TFOTs) and Pressure Aging Vessel (PAV) aging tests were conducted, followed by dynamic shear rheology (DSR) tests, to study the high-temperature rheological properties of each warm mix HMA. Fourier-transform infrared spectroscopy (FTIR) tests and fluorescence microscopy were used to observe the microstructures of the asphalt. The results show that all six wax materials exhibited good warm mix effects, among which refined Fischer–Tropsch Wax 1 (RFW1) outperforms conventional Sasobit WMA in terms of warm mix effect, high-temperature rheological properties, and anti-aging performance, indicating its potential to replace Sasobit in engineering applications.

## 1. Introduction

Road infrastructure plays a major role in the economic development of countries around the world as an important asset of each country [1]. As the global road transportation network continues to grow, so does the demand for high-performance asphalt mixtures [2,3,4]. Therefore, HMA is mostly used in road construction [5]. SBS block copolymers are widely used high-viscosity modifiers, which can greatly improve the physical properties and rheological properties of asphalt [6].

Many researchers have studied HMA, including its low-temperature performance, high-temperature performance, anti-aging performance, microstructure, and so on. Zhang et al. [7] used FTIR and fluorescence microscopy to study the effects of different aging conditions on the performance of SBS copolymer under the action of penetration on 70# and 90# base asphalt grades, and the results showed the following: SBS-90 aged more markedly than SBS-70, and UV aging more significantly degraded SBS copolymer compared to PAV aging. Li et al. [8] investigated the basic properties and thermal stability of HMA under the action of two new WMAs, and the results showed that HMA showed improvements in construction, ease of construction and low-temperature performance under the action of the two kinds of WMA, respectively. Therefore, they believe that HMA can provide a better performance when applied to asphalt pavements in cold regions under the action of these two warm mixes. Tian et al. [9] studied the properties of matrix asphalt under SBS and polyurethane prepolymer (PUP)-compliant modification via Fourier-transform infrared spectroscopy experiments, repeated stress creep recovery tests, stability tests, etc., and they found that SBS/PUP composite modified asphalt has a good phase structure, storage stability, and high-temperature performance, and it is suitable for demanding working environments. Xing et al. [10] studied the impact of TFOTs, PAV tests, and humid-heat cyclic aging on the performance of base asphalt and three kinds of HMA through Fourier-transform infrared spectroscopy, thermogravimetric analysis, and so on. The test results showed that the wet-heat cycle accelerates the aging of HMA and shortens its service life.

However, traditional hot-mix asphalt mixtures need to be heated at high temperatures during production and construction, which consumes large amounts of energy and emits large amounts of harmful gases [11,12,13]. As an energy-saving and environmentally friendly new asphalt mixing technology, warm mix asphalt can be mixed and constructed at relatively low temperatures, effectively reducing energy consumption and harmful gas emissions [14,15], which can help China to realize the goal of “double carbon”. Wax WMA has a significant warm mixing effect and is a commonly used type of WMA in warm mixing asphalt technology, as it has unique physical and chemical properties and can reduce the viscosity of asphalt at lower temperatures to realize the warm mixing effect [16,17,18]. WMA is a broad family of technologies aimed at reducing production and compaction temperatures. In addition to wax-based additives, WMA technologies also include bitumen foaming techniques (e.g., water/zeolite-assisted foaming) and chemical additives (e.g., surfactants, adhesion promoters, and viscosity modifiers) that improve coating and workability at reduced temperatures. These approaches have been widely discussed in the literature and provide important context for positioning wax-based WMA within the overall WMA technology landscape. In this study, we focus on wax-based additives because they can be applied directly to the binder and can simultaneously influence construction viscosity and in-service rheology through crystallization-related mechanisms. Moreover, compared with other types of warm mixes, wax WMAs may have a cost advantage in some cases, which is conducive to the promotion and application of warm mix asphalt technology. Among wax-based WMA additives, Sasobit is a widely used commercial WMA product and is frequently adopted as a benchmark in laboratory studies. It is commonly categorized as a Fischer–Tropsch (FT) wax product. It primarily consists of long-chain, predominantly linear aliphatic hydrocarbons (n-alkanes) with a broad carbon-number distribution, and it typically exhibits a melting/transition range around 100~110 °C (with a nominal melting point reported near 104 °C, depending on the product grade). Therefore, in this work, Sasobit was selected as the reference wax additive, and several alternative wax products with different chemical features were evaluated under identical preparation and testing conditions, but HMA is prone to aging under high-temperature conditions, and its performance is unstable (partially summarized in Table 1). In addition, the use cost of Sasobit is higher, as a new type of wax material can replace Sasobit WMA and does not affect the performance of HMA. This is of great significance for optimizing the production process and performance of warm mix HMA.

Despite the growing body of work on wax-based WMA additives, an unresolved scientific issue remains: how the molecular architecture of a wax (e.g., carbon-number distribution, linearity/branching, and the presence of oxygen- or nitrogen-containing functionalities) governs the coupled performance of SBS HMA in terms of viscosity reduction at construction temperatures and structural stability against thermo-oxidative aging. Most prior studies have focused on commercial Fischer–Tropsch (FT) waxes such as Sasobit, while refined FT waxes with different compositional fingerprints have been far less explored under the same base binder, SBS content, and dosage-controlled conditions. The purpose of this study is to explore the introduction of wax materials to try to replace the traditional Sasobit WMA without affecting the aging performance of HMA. Five new wax materials were introduced in the research process: modified polyamide wax (MPW), refined Fischer–Tropsch wax 1 (RFW1), refined Fischer–Tropsch wax 2 (RFW2), special wax (SW), oxidized polyethylene wax (OPW). Firstly, the rheological properties of HMA under the action of six wax materials and HMA without wax materials under non-aging, short-term aging (TFOT) and long-term aging (PAV) conditions were studied via DSR. Secondly, the changes in the chemical structure of seven groups of asphalt under non-aging, TFOT aging and PAV aging conditions were studied using FTIR. Finally, the micro-morphological changes in seven groups of asphalt under non-aging, TFOT aging and PAV aging conditions were observed via fluorescence (FM) microscope testing, so as to optimize the wax WMA with a better warm mixing effect and more stable performance of HMA.

## 2. Material and Methods

### 2.1. Test Materials

#### 2.1.1. Asphalt

The high-viscosity asphalt samples were prepared from SK base asphalt produced from a Korean asphalt plant with penetration of 60/80, and the properties are shown in Table 2.

#### 2.1.2. SBS Modifier

The ratio of styrene/butadiene for SBS is 32/68. In this study, the content of SBS in the preparation of HMA is 5.2% [23]. The basic properties of SBS modifier are shown in Table 3.

#### 2.1.3. Wax Materials

**Figure 1:**
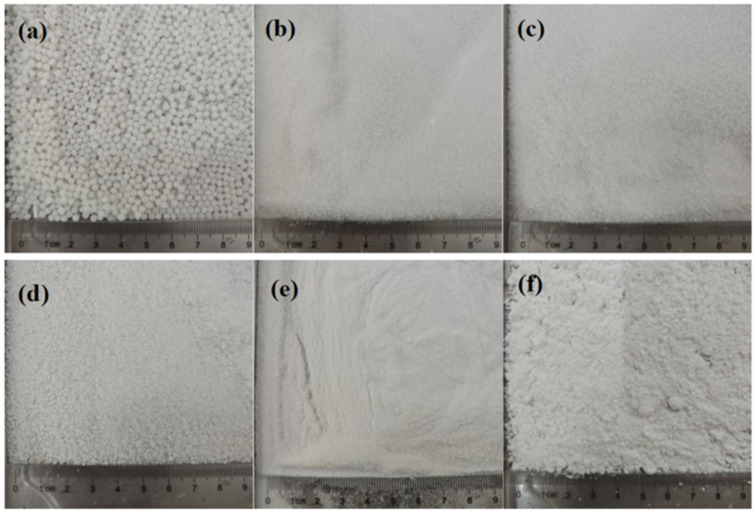
Appearance of warm-mixed additives: (**a**) Sasobit; (**b**) RFW1; (**c**) RFW2; (**d**) MPW; (**e**) OPW; (**f**) SW.

The wax materials used in this study are as follows: Sasobit, MPW, RFW1, RFW2, SW, and OPW. Images of these six wax materials are shown in Figure 1, and basic information is given in Table 4.

### 2.2. Material Preparation and Evaluation Methods

#### 2.2.1. Preparation of Warm Mix HMA

**Figure 2:**
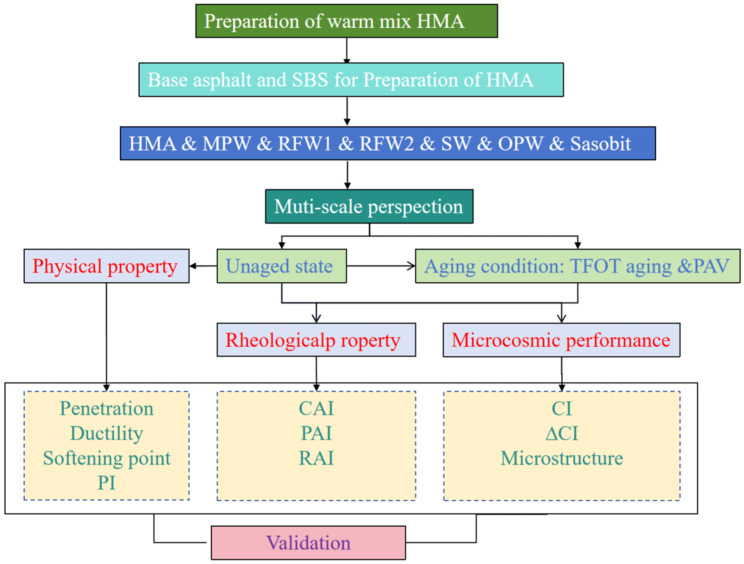
Experimental methodologies and research pathways employed in this study.

In this study, HMA was first prepared using externally doped 5.2% SBS high-viscosity modifier with base asphalt. During the preparation process, the asphalt was heated to 175 °C. After adding the SBS high-viscosity modifier, the rotational speed of the shear was adjusted to 4000 rpm, and the shear was performed for 40 min. Then, the produced HMA was divided into seven portions, and the six waxes mentioned before were added into six of them, respectively, and stirred with a stirrer at 175 °C for 2 h; the remaining portion was left as a blank sample. The specific preparation process is shown in Figure 2. Referring to existing studies, the optimal content of Sasobit in HMA is 3% [27,28]. A dosage of 3 wt.% (by mass of binder) was used for all wax additives to enable a controlled, head-to-head comparison under a unified dosage level, minimizing confounding effects from dosage variability. This value was selected based on published practice for wax-based WMA additives and, in particular, on reports indicating that 3% is an effective and commonly adopted dosage for Sasobit in SBS-modified and/or high-viscosity binders, providing a meaningful benchmark for warm mix effectiveness. Using the same dosage for the alternative wax products allows differences in viscosity–temperature behavior, rheology, aging resistance, and microstructure to be primarily attributed to wax chemistry and phase-transition characteristics, rather than to different dosage levels. Therefore, the content of the remaining five wax materials was set at 3% for parallel control. The seven HMAs were named blank sample, S-Sasobit, S-MPW, S-RFW1, S-RFW2, S-SW, and S-OPW. To ensure no severe oxidative degradation of SBS occurred after preparation, we conducted qualitative verification via fluorescence microscopy. The SBS-rich phase exhibited the expected network/continuous morphology in the unaged adhesive, with consistent rheological responses across all replicate batches. Furthermore, FTIR spectra showed no new absorption bands indicative of main-chain chemical transformations.

#### 2.2.2. Basic Performance Test

In order to preliminarily evaluate the basic physical properties of the six prepared warm-mixed HMA s and the blank control group of HMA s without warm additives, penetration (25 °C), softening point, and elongation (5 °C) tests were conducted in this study according to the ASTM and AASHTO specifications for modified asphalts. Penetration index (PI) can be used to assess the temperature sensitivity of asphalt; the higher the PI, the lower the temperature sensitivity of asphalt. When the PI value is greater than or equal to 2, the asphalt will have a gelled structure; when the PI value is less than 2, the asphalt will have a sol–gel structure. The formula for calculating the PI value is shown in Equation (1):

(1) $$PI\;=\;\frac{1952\;-\;500\log\left(P_{25}\right)-20S_{p}}{50\log\left(P_{25}\right)-\;S_{p}\;-\;120}$$

where S_p_ is the softening point; $P_{25}$ is the degree of penetration at 25 °C.

#### 2.2.3. Rheological Properties Test

**Figure 3:**
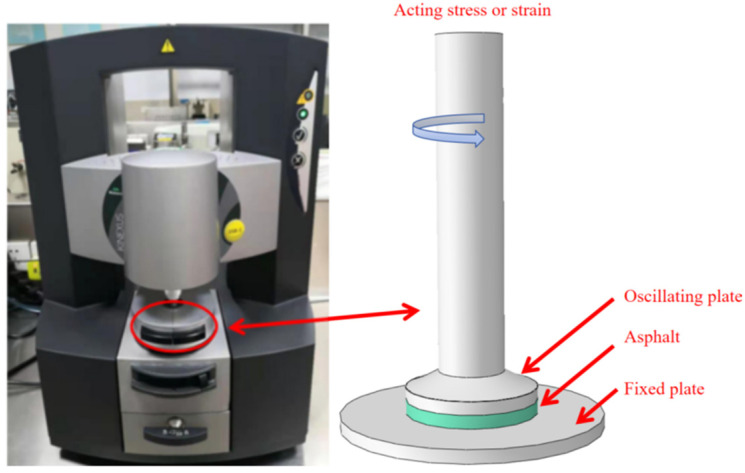
Asphalt rheological properties test.

The complex shear modulus and phase angle can reflect the rheological properties of asphalt, and a dynamic shear rheometer can measure the complex shear modulus and phase angle of asphalt by applying oscillatory shear stress or strain. Therefore, dynamic shear rheology testing is widely used in the fields of high-temperature performance evaluation and aging performance analysis of asphalt. In this study, the rheological properties of seven groups of asphalts before and after aging are investigated according to the AASHTO specification. Specifically, the seven unaged groups of asphalt, as well as the seven groups of asphalt after short-term aging, long-term aging, and each of the samples, were taken for temperature scanning tests (Figure 3). The test was conducted using strain-controlled loading, with a set plate gap of 1 mm and a diameter of 25 mm. The control strain was 12%, and the oscillation angle frequency was 10 rad/s. The strain level was selected to obtain stable torque signals across high-temperature ranges and improve the repeatability of high-viscosity SBS-modified adhesives. To ensure the chosen strain amplitude remained within a reasonable range, representative temperatures were selected within the temperature scanning range, and preliminary strain scanning tests were conducted using the same loading frequency as the temperature scan. The results confirmed that the complex modulus, phase angle, and rutting resistance factor at 12% strain conditions all corresponded to linear viscoelastic responses. In a temperature interval from 46 to 82 °C, the first temperature scan was conducted at 46 °C, and thereafter, temperature scans were conducted once for every 6 °C rise in temperature, for a total of seven times. The high-temperature performance evaluation index is chosen as the complex modulus (G*), phase angle (δ) and rutting factor (G*/sin δ), where G* and δ characterize the viscous and elastic properties of asphalt, respectively, and (G*/sin δ) characterizes the rutting resistance of asphalt mixtures.

#### 2.2.4. Aging Process

In order to evaluate the effect of WMA on the performance of HMA after aging, short-term aging (TFOT) and long-term aging (PAV) tests were conducted on seven groups of asphalt according to ASTM D1754 [29] and ASTM D6521 [30] specifications, respectively. During TFOT aging, the samples were placed in containers at 163 °C for 85 min; during PAV aging, the samples were placed in glass aging vessels at 100 °C for 20 h. In this study, the changes in rheological indexes before and after aging of warm-mixed HVMA were used to evaluate the aging sensitivity, which is defined.

Complex modulus aging index (CAI):

CAI = G*_aged_/G*_unaged_ (2)

Phase angle aging index (PAI):

PAI = δ_aged_/δ_unaged_ (3)

Rutting factor aging index (RAI):

RAI = |(G*/sin δ_aged_ − G*/sin δ_unaged_)| × 100/G*/sin δ_unaged_ (4)

#### 2.2.5. FTIR Experiment

The aging of asphalt is usually caused by factors such as oxidation, volatile losses, and temperature changes, among which thermal oxidation is the main cause of asphalt aging in high-temperature environments [31]. During this process, the number of functional groups such as carbonyl and sulfinyl groups in asphalt increases or decreases [32]. In this study, FTIR was used to observe the changes in carbonyl content before and after aging of warm-mixed HMA and to determine the degree of aging of asphalt. That is, the carbonyl index (CI) was used to characterize the thermal aging degree of asphalt. The larger the difference in CI (∆CI) before and after aging of warm-mixed HMA, the deeper the degree of aging, and the definition of CI is as follows:

(5) $$CI=\frac{A_{C=O}}{\sum A}$$

where $A_{C=O}$ is the area of the absorption peak of the carbonyl functional group (C=O) at a wave number of 1700 cm^−1^; ∑A is the sum of the areas of the absorption peaks, $\sum A=A_{1700cm^{-1}}+A_{1600cm^{-1}}+A_{1460cm^{-1}}+A_{1376cm^{-1}}+A_{1030cm^{-1}}+A_{864cm^{-1}}+A_{814cm^{-1}}+A_{743cm^{-1}}+A_{724cm^{-1}}$. In this study, the warm-mixed HMA samples were first placed on the FTIR experimental bench, compacted, and fixed with a metal layer. Then, the wave number range was set from 4000 cm^−1^ to 400 cm^−1^, 32 scans were performed, and the average of the scans of the respective samples was taken. Finally, we utilized the 2021 version of MATLAB to analyze the spectra.

#### 2.2.6. FM Experiment

SBS modifier in asphalt undergoes a fluorescence reaction [33]. To study the effect of the type of wax material on its dispersion effect and microstructure, the microstructural morphology of seven groups of asphalt after unaged, short-term aging and long-term aging was observed using a fluorescence microscope in this study to evaluate the warm mixing effect of each wax material. For FM test, each group of asphalt was dripped on the slide to make a thin film of asphalt and then examined under a 10× fluorescence microscope.

## 3. Results and Discussion

### 3.1. Basic Performance Analysis

**Figure 4:**
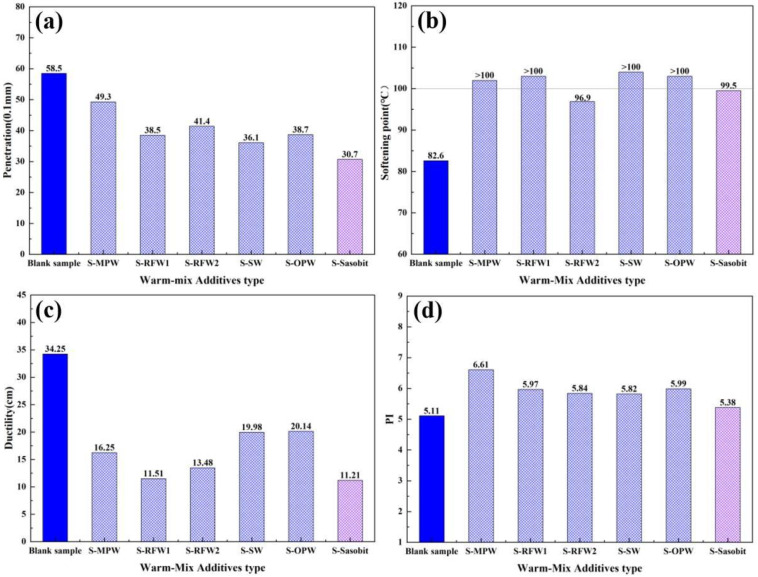
Effect of different WMAs on the basic properties of HMAs: (**a**) penetration; (**b**) softening point; (**c**) ductility; (**d**) PI.

Figure 4a shows the penetration of different warm-mixed HMAs, from which it can be seen that the penetration of HMAs is reduced by the addition of waxes, and the reduction in Sasobit is the most obvious, reaching 47.5%. This may be due to the fact that waxes partially crystallize at 25 °C, which can have the effect of hardening the asphalt binding material. According to the softening point test data in Figure 4b, it can be seen that each wax material has the effect of increasing the softening point of HMA, among which RFW2 and Sasobit have poorer effects than the other four types of wax materials. The ductility reflects the low-temperature performance of asphalt. The higher the 5 °C ductility, the better the low-temperature performance of asphalt, as shown in Figure 4c. The addition of wax materials reduced the ductility of HMA to varying degrees, in which SW and OPW have a relatively small effect on the low-temperature performance of asphalt, respectively, reduced by 41.97% and 41.2%. From Figure 4d, PI can be seen: all six wax materials increased the penetration index (PI) of the SHVA, reduced the temperature sensitivity of the asphalt, and the PI of the SHVA changed most significantly after the addition of MPW, with an increase of 29.4% in the PI.

### 3.2. DSR Test

#### 3.2.1. DSR Analysis of Experimental Results

**Figure 5:**
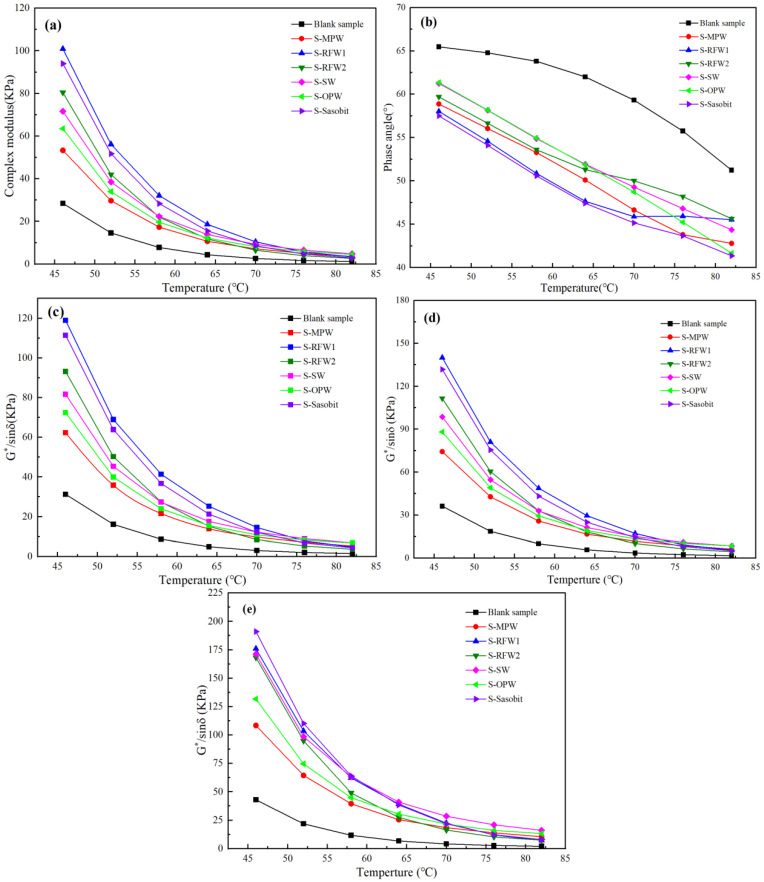
(**a**) Complex modulus values of unaged groups of asphalts; (**b**) phase angles of unaged groups of asphalts; (**c**) rutting factor values of unaged groups of asphalts; (**d**) rutting factor values of groups of asphalts after short-term aging; (**e**) rutting factor values of groups of asphalts after long-term aging.

The complex modulus of asphalt is used to characterize its resistance to deformation, and the phase angle is an indicator of asphalt viscosity to elastic component ratio asphalt. Also, larger asphalt values represent greater resistance to high temperature [34]. The values of complex modulus, phase angle, and rutting resistance factor for each group of asphalt when unaged are shown in Figure 5. As can be seen from Figs. a, b, relative to the blank group, the addition of all six wax materials increases the complex modulus of the HMA while decreasing the phase angle, i.e., decreasing the viscosity of the HMA while increasing its elastic properties. This may be since, when the temperature is lower than the melting point of the wax material, it will exist in the asphalt binder in the form of reticulated crystals, which can enhance the intermolecular bonding and make the asphalt more resistant to deformation [35]. At the same time, waxes generally have a high degree of crystallinity and rigidity, and when added to asphalt, they increase the elastic component of the asphalt and relatively reduce the viscous component, which leads to a decrease in the phase angle. According to the Superpave specification for grading asphalt binder properties, it is known that for unaged asphalt, the temperature corresponding to G*/sinδ = 1.0 KPa is defined as the critical temperature, and the higher the critical temperature, the better the asphalt’s high-temperature performance. It can be seen in Figure 5c that the critical temperature of asphalt under the action of each wax material will be greater than 82 °C, and the rutting resistance factor in the range of 46~82 °C is numerically greater than that of the blank group. In terms of the effect of improving the performance of HMA, RFW1 > Sasobit > RFW2 > SW > OPW > MPW. In addition, Figure 5d,e show the trend of the rutting factor with temperature for each group of asphalt after short- and long-term aging. It can be seen from the figures that the addition of wax materials can also improve the high-temperature rutting resistance of HMA after short- and long-term aging relative to the blank sample.

#### 3.2.2. Rheological Aging Index Analysis

**Figure 6:**
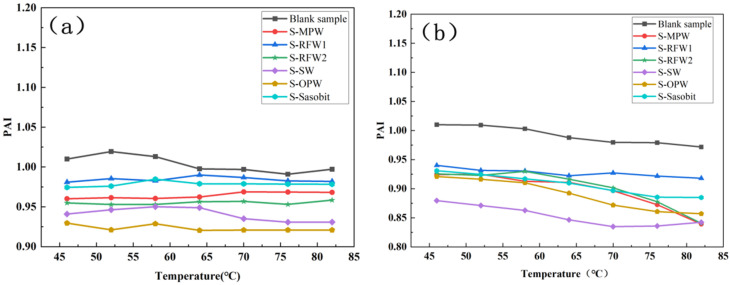
PAI of different warm mix HMA after TFOT and PAV: (**a**) TFOT; (**b**) PAV.

Figure 6 demonstrates the magnitude of PAI at different temperatures for HMA under the action of different wax materials after short- and long-term aging. Comparing the results of the two aging methods, it can be found that the PAI values of each group of asphalt after PAV aging are correspondingly smaller than those after TFOT aging, which is because long-term aging will lead to a more obvious decrease in the content of the viscous component in the asphalt, and so the asphalt will have a smaller phase angle. Comparing the blank samples, it can be found that the PAI values of the HMA under the effect of different wax materials are significantly smaller after TFOT and PAV aging. In general, the PAI of asphalt under the action of RFW1 and Sasobit decreased the least relative to the blank samples, regardless of TFOT aging or PAV aging. However, according to Figure 6b, the PAI value of Sasobit was slightly lower than that of RFW2 in the range of 52–70 °C. The PAI value of Sasobit was also slightly lower than that of RFW2 [36].

**Figure 7:**
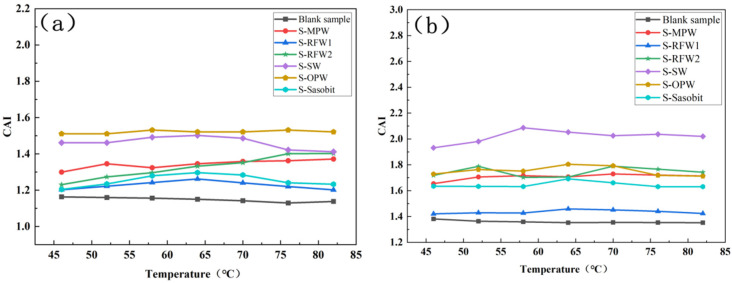
CAI of different warm mix HMA: (**a**) TFOT; (**b**) PAV.

Figure 7 shows the changes in the CAI of seven groups of asphalts after TFOT and PAV aging, respectively. Compared with the blank samples, the CAI values of the asphalts under the action of the six wax materials are significantly larger than those of the blank samples. In general, the asphalt under the action of RFW1 and Sasobit has relatively small CAI values, while the asphalt under the action of SW and OPW has relatively large CAI values, which is basically consistent with the calculation results of PAI. This is due to the different main compositions of the wax materials and the different mechanisms of interaction between each of them and the asphalt.

**Figure 8:**
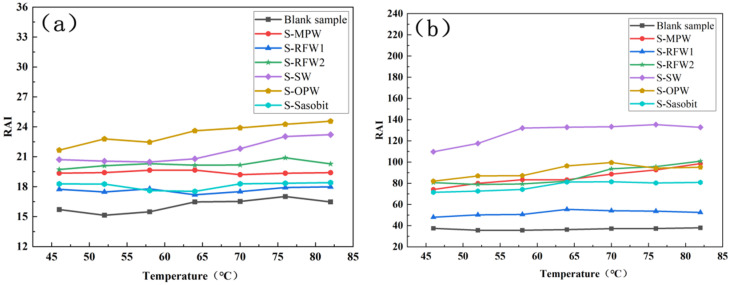
RAI of different warm mix high-viscosity asphalt: (**a**) TFOT; (**b**) PAV.

After TFOT and PAV aging, the changes in the RAI values of the seven groups of asphalts are shown in Figure 8. From the figure, it can be found that after PAV aging, the RAI values of all groups of asphalts are significantly larger than the RAI values of TFOT aging, especially the HMA under SW. The fact that the difference between the RAI values of each group of waxed warm mix asphalt under the two aging modes is so obvious indicates that the mode of thermal oxidation has a greater impact on the high-temperature rutting resistance of waxed warm mix asphalt. In addition, in general, comparing with the blank samples, the RAI values of asphalts under SW and OPW in both aging modes are significantly larger than those of the blank samples, while the asphalts under RFW1 and Sasobit still have RAI values with relatively small increases [37]. This indicates that RFW1 and Sasobit have the least effect on the aging resistance of HMA among the six wax materials. In summary, the HMA under the effect of RFW1 has relatively good rheological properties, and the effect of RFW1 on the aging resistance of HMA is minimized, whether analyzed from the point of view of CAI, PAI or RAI.

### 3.3. FTIR Test

#### 3.3.1. Qualitative Inorganic Analysis

**Figure 9:**
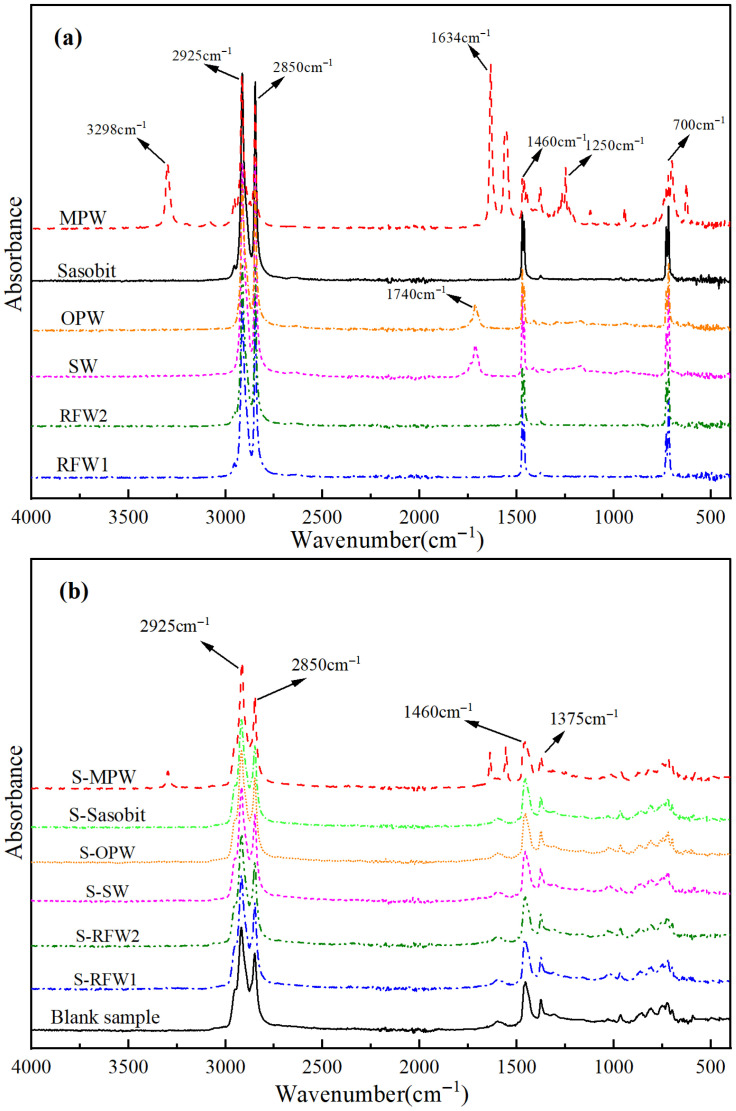
Infrared spectra of each wax material: (**a**) six kinds of wax materials; (**b**) warm mix modified asphalt.

The infrared spectra of the six wax materials are shown in Figure 9a, from which it can be seen that the positions of the absorption peaks appearing in the infrared spectra of Sasobit, RFW1 and RFW2 are basically the same, and their main absorption peaks appear due to the C-H bond stretching vibrations in the methyl and methylene groups of the alkanes, i.e., at wavelengths of 2925 cm^−1^ and 2850 cm^−1^, and the bending vibrations of aliphatic CH_2_ and aromatic C-H at wavelengths of 1460 cm^−1^ and 700 cm^−1^ at aliphatic CH_2_ and aromatic C-H bending vibrations. This indicates that the main components of these three wax materials are basically similar to the asphalt fractions. In addition, there is a special absorption peak at 1740 cm^−1^ in the infrared spectra of SW and OPW, which is caused by the stretching vibration of the polar group carbonyl (-C=O). This indicates the presence of not only nonpolar but also polar components in these two wax materials. And there are also two special absorption peaks at wave numbers of 1634 cm^−1^ and 3298 cm^−1^ for MPW, which correspond to the deformation vibration and stretching vibration of the amide group (-NH-), respectively. Figure 9b shows the infrared spectra of the blank sample and each wax material after being added to the HMA. From the figure, it can be found that the locations of the absorption peaks appearing in the asphalt under the action of Sasobit, RFW1, RFW2, SW and OPW are basically the same as that of the blank sample, and no new absorption peaks are generated. And, compared with Figure 9a, it can be found that the additional absorption peaks of the asphalt under the action of MPW are caused by the special functional groups of the MPW materials themselves, i.e., no new absorption peaks are generated. Therefore, it can be assumed that the six wax materials have good compatibility with the HMA and will not have a chemical reaction with the asphalt or SBS modifier when added to the HMA, and only physical fusion exists, i.e., the warm mixing mechanism of the six wax materials does not involve the chemical reaction process. It should be noted that some absorption peaks in the spectra of the wax materials disappear in the asphalt spectra; for example, the absorption peaks of OPW and SW at 1740 cm^−1^ do not exist in the HMA, which may be because the molecules of the wax materials will be relatively dispersed in the asphalt; i.e., the molecules located in the environment have changed.

**Figure 10:**
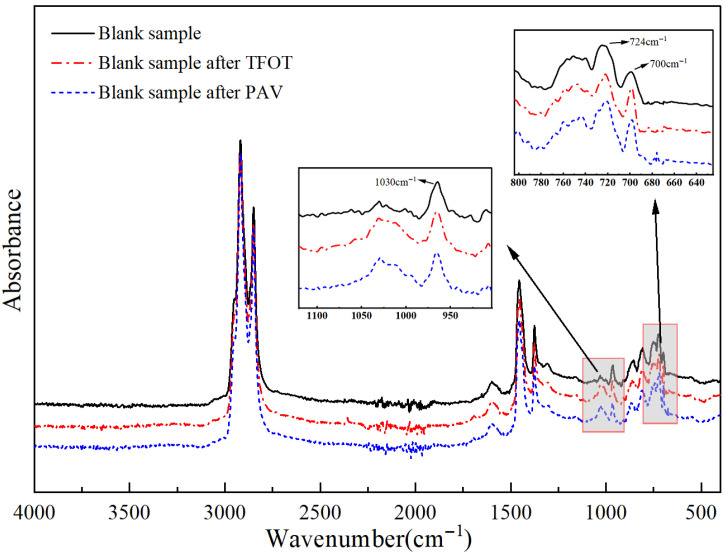
Infrared spectra of HMA before and after aging.

Figure 10 shows the infrared spectra of HMA after TFOT and PAV aging. As can be seen from the figure, in the asphalt after aging in the infrared spectrum at 1030 cm^−1^, the intensity of the absorption peaks increased significantly, corresponding to the sulfinyl group. Asphalt in the generation of sulfinyl is one of the important symbols of the aging of asphalt [38], so it can be determined that the TFOT and PAV aging method of the asphalt composition was caused by the impact. In addition, in HMA before and after aging, there will be an absorption peak intensity-weakening phenomenon, for example, the wave number of 690~750 cm^−1^, which indicates that aging will reduce the asphalt in the light component content of the asphalt. From the two aging methods of asphalt absorption peaks, the longer the aging time, the more the light component content decreases.

**Figure 11:**
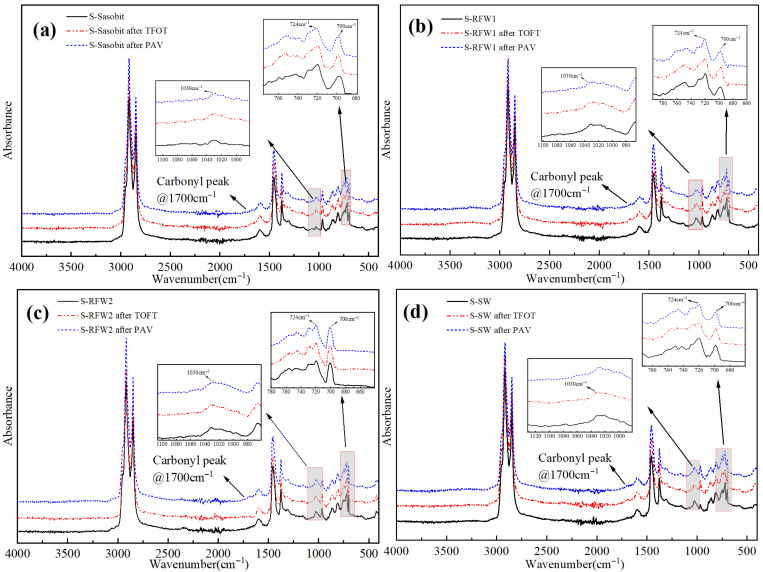

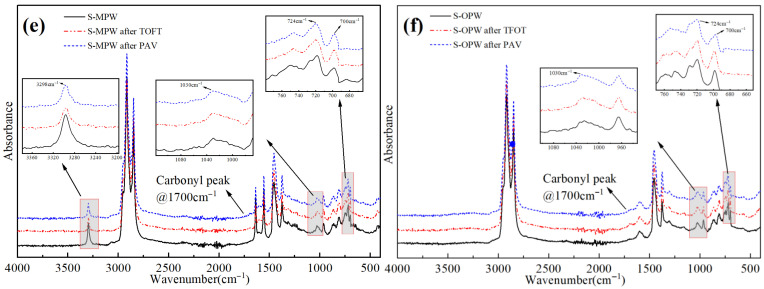
Infrared spectra of HMA before and after aging in the presence of six wax materials: (**a**) Sasobit; (**b**) RFW1; (**c**) RFW2; (**d**) SW; (**e**) MPW; (**f**) OPW.

Figure 11 demonstrates the IR spectra of HMA before and after aging in the presence of wax materials, respectively. As mentioned earlier, the wave number in the IR spectrum representing the light component content in the asphalt is 724~868 cm^−1^, and observing the IR spectra of the asphalt under the action of Sasobit, RFW1 and RFW2, it can be found that the decrease in the absorption peak area in this wave number range is smaller than that of the other three wax materials. This may be due to the fact that, although Sasobit, RFW1 and RFW2 are different Fischer–Tropsch waxes, their main components are straight-chain alkanes, which are more compatible with the saturated fraction of asphalt, and the dispersion uniformity is higher. When the asphalt is cooled down, it can rapidly form microcrystalline lattices to hinder the diffusion of oxygen, thus slowing down the aging rate. Moreover, compared with oxygenated waxes (OPW), straight-chain alkanes are more resistant to oxidation due to their simple structure and higher bond energies (C-C bond energy 347 KJ/mol, C-H bond energy 413 KJ/mol). In addition, the infrared spectra of asphalt under the action of MPW show a smaller decrease in the absorption peak area in this wavelength range compared to OPW and SW, which may be due to the fact that OPW and SW both have high melting points and polar components, resulting in poor compatibility between the two and the saturated and aromatic components of the asphalt, the occurrence of phase separation in aging, and microcrystalline structure collapse, so the decrease in the light component content is larger. On the other hand, the amide bond, which is unique to MPW, is resistant to high temperatures and can maintain the microcrystalline structure through hydrogen bonding [39].

#### 3.3.2. Quantitative Analysis

**Figure 12:**
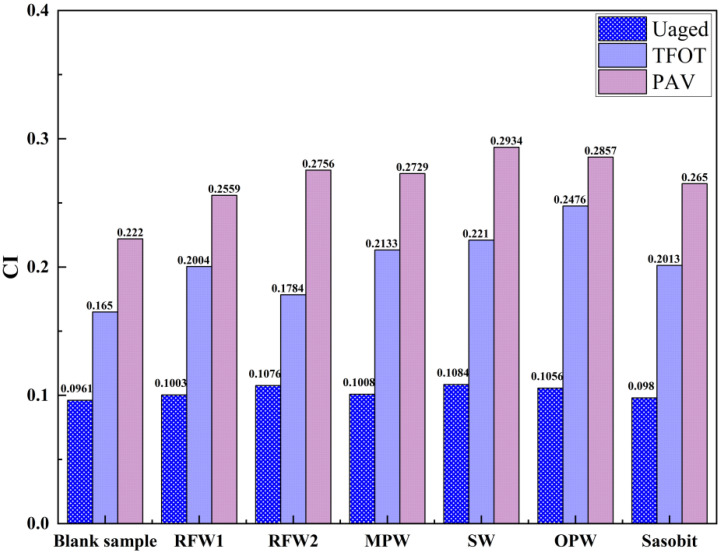
Carbonyl index of warm mix HMA under different aging conditions.

In order to further analyze the changes in functional groups before and after aging of each group of asphalt, the carbonyl index (CI) was introduced to quantitatively analyze the warm mixing effect of each wax material. The carbonyl index, indicative of the relative content of carbonyl groups in asphalt, serves as a key marker for the polar components within the asphalt matrix. A higher carbonyl index signifies stronger intermolecular interactions in the asphalt, which, in turn, translates to enhanced high-temperature stability [40]. The CI and ∆CI values of each group of asphalts before and after aging are shown in Figure 12 and Table 5. From Figure 12, it can be seen that all groups of HMAs show an increase in CI after short-term aging and long-term aging. And, comparing the aging results of TFOT and PAV, it can be found that the longer the aging time, the greater the CI, i.e., the deeper the degree of thermal oxidation of asphalt. In addition, comparing other wax materials, the HMA under RFW1 and Sasobit has relatively smaller CI values after short- and long-term aging. This indicates that whether it is TFOT aging or PAV aging, the HMA under the action of RFW1 and Sasobit has the lowest degree of oxidative hardening compared to other wax materials. Also, observing Table 5, it can be seen that after short-term aging, the size order of ∆CI of each group of asphalt is S-OPW > S-SW > S-MPW > S-Sasobit > S-RFW1 > S-RFW2 > blank sample, and after long-term aging, the size order of ∆CI of each group of asphalt is SW > OPW > MPW > RFW2 > Sasobit > RFW1 > blank sample. This shows that under short-term aging conditions, RFW1 and RFW2 have better anti-aging protection effects on HMA relative to other wax materials, while under long-term aging conditions, RFW1 and Sasobit have better anti-aging protection effects on HMA.

### 3.4. FM Test

**Figure 13:**
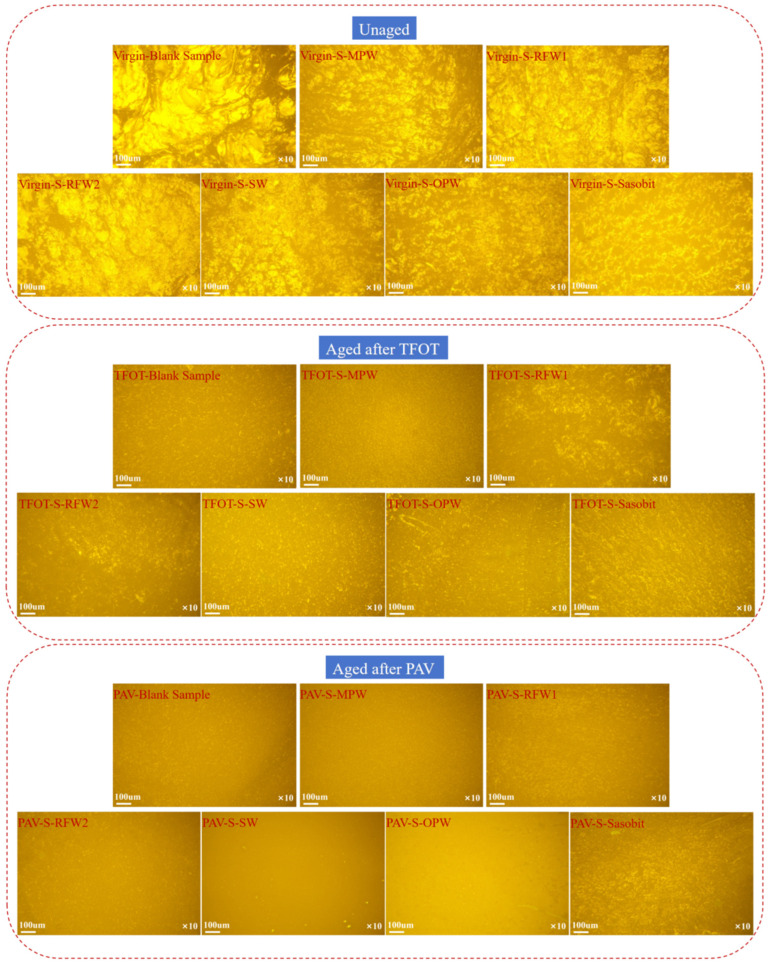
Micro-morphology of HMA under the action of different wax materials in different aging states.

In this study, the microstructures of seven groups of asphalt after unaged, short-term aging and long-term aging were observed using a fluorescence microscope, and the microstructures of each asphalt were obtained. From Figure 13, for each group of asphalt in the unaged state, compared with the blank samples, it can be found that the addition of wax materials can significantly improve the agglomeration phenomenon of SBS modifier in the asphalt, so that it is more uniformly distributed in the asphalt. This is because wax materials mixed in asphalt can play a similar role to lubricants. Under identical sample preparation and imaging conditions, the fluorescence micrographs suggest a more uniform distribution of the SBS-rich fluorescent phase in the RFW1-, RFW2-, and Sasobit-modified binders, with fewer visually apparently large agglomerates compared with the blank binder and the other wax-modified binders. This may be due to the fact that these three are straight-chain alkanes, which are similar to the main components of asphalt, and, therefore, have relatively better compatibility and uniformity of dispersion with asphalt.

Comparing the microstates of asphalt under unaged and short-term aging conditions, it can be found that the reticular, filamentary and punctate fluorescence in the images was reduced under short-term aging conditions. This suggests that short-term aging causes structural damage and degradation of SBS modifiers. The structural damage to the SBS modifier in HMA under MPW, SW and OPW is more serious. On the other hand, the structural damage to the SBS modifier in asphalt under RFW1, RFW2 and Sasobit is relatively small and better than the morphology of blank samples after short-term aging. This may be due to the fact that Fischer–Tropsch wax has a high crystallinity and melting temperature, which can physically impede the contact of oxygen with asphalt molecules, thus maintaining better performance during short-term aging [41].

Observation and comparison of the micro-morphology of each group of asphalt under the unaged and long-term aging conditions show that the fluorescent components in each group of asphalt basically disappeared after long-term aging. This indicates that long-term aging will substantially damage or degrade the network and other structures formed by the SBS modifier in the HMA. Only from Sasobit and RFW1 under the action of HMA micrographs can a relatively obvious SBS modifier-formed structure be seen. The other four wax materials and the blank samples after long-term aging can hardly be seen. This may be due to the fact that the three-dimensional polymer network formed by SBS in asphalt is interspersed with the crystalline structures of RFW1 and Sasobit, which can effectively inhibit the thermal movement of SBS molecular chains during long-term thermo-oxidative cycling, reducing phase segregation and oxidative chain breakage due to thermal stress.

## 4. Conclusions

In this study, five kinds of wax materials were introduced to try to replace the traditional Sasobit WMA to weaken the aging performance of HMA under high-temperature conditions to optimize the wax WMA with better warm mix effects and more stable performance. According to the above experimental study, the following conclusions are drawn:

(1) Six kinds of wax materials can improve the rheological properties of HMA, and from the effect point of view, RFW1 > Sasobit > RFW2 > SW > OPW > MPW. After TFOT and PAV aging, the anti-rutting performance of HMA under the action of RFW1 and Sasobit is relatively good. In addition, from the three aging indexes (CAI, PAI and RAI), RFW1 has the least influence on the anti-aging performance of HMA.
(2) There is no chemical reaction between wax materials and HMA. After short-term aging, the order of ΔCI for each group of asphalt is as follows: OPW > SW > MPW > Sasobit > RFW1 > RFW2 > blank sample. After long-term aging, the order of ΔCI is SW > OPW > MPW > RFW2 > Sasobit > RFW1 > blank sample. Under short-term aging conditions, RFW1 and RFW2 have little effect on the aging performance of HMA. Under long-term aging conditions, RFW1 and Sasobit have little effect on the aging performance of HMA.
(3) Under short-term and long-term aging, RFW1, RFW2 and Sasobit have a relatively small effect on the dispersion uniformity of the SBS modifier in HMA, and they are better than the morphology of the blank samples after short-term aging.
(4) Under the action of RFW1 wax material, HMA showed excellent rheological properties and anti-aging properties. FM testing also confirmed this from the microscopic properties. Therefore, we recommend the use of RFW1 in the warm mix HVMA process.

## Figures and Tables

**Table 1:** Existing studies on the effect of different WMAs on the performance of HMA.

Reference	Field of Research	Relevant Results
Xingmin Liang et al. [19]	Effect of surfactant Evotherm, foaming water, wax warm mix Sasobit and EC120 on the properties of HMA.	Sasobit, EC120, and foam water adversely affected the medium temperature fatigue properties and low-temperature crack resistance of HMA.
Bei Chen et al. [20]	Effect of Foaming Water, Wax Warm Mix Sasobit and Surfactants (Evotherm and New Warm Mix GLWBR) on the Performance of HMA.	All four warm mixes were able to achieve a warm mix effect, with Foam Water and Sasobit having the best warm mix effect.
Jie Gong et al. [21]	Effect of Sasobit concentration on morphology and properties of epoxy HMA.	Sasobit disrupts the spherical phase separation morphology and increases the Young’s modulus of epoxy HMA.
Wenhao Dong et al. [22]	Effect of Sasobit, Evotherm and new organic composite warm mixes on the performance of HMA.	HMA in the presence of new organic composite warm mixes showed better low temperature performance compared to Sasobit and Evotherm.

**Table 2:** Properties of matrix asphalt.

Inspection Index	Unit	Quality Index	Result
Penetration	0.1 mm	60–80	68
Softening point	°C	≥46	47.8
Ductility (15 °C, 5 cm/min)	cm	≥20	>100
Dynamic viscosity 60 °C	Pa∙s	≥180	167.5
Density (15 °C)	g/cm^−3^	-	1.047
Flash point	°C	≥260	345

**Table 3:** The basic properties of SBS.

Parameter	Results	Standards
Proportion/g cm^−3^	0.94	ISO 2781 [24]
Melt flow index/g∙10 min^−1^	6	ASTM D1238 [25]
Tensile strength/MPa	160	ASTM D412 [26]
Elongation/%	680	ASTM D412

**Table 4:** Basic information on the six types of WMAs with waxes.

Category	Morphology	Melting Point (°C)	Production Company
Sasobit	White particles	104	Sasol Group, Johannesburg, South Africa
RFW2	White beads	110	Tianshi, Nanjing, China
SW	Yellowish fine powder	137	Tianshi, Nanjing, China
OPW	Pale yellow particles	137	Tianshi, Nanjing, China
MPW	White particles	145	Tianshi, Nanjing, China
RFW1	White beads	112	Tianshi, Nanjing, China

**Table 5:** Incremental carbonyl index (∆CI) of warm mix HMA under different aging conditions.

Sample	Blank Sample	S-RFW1	S-RFW2	S-MPW	S-SW	S-OPW	S-Sasobit
TFOT	0.0689	0.1001	0.0708	0.1125	0.1126	0.142	0.1033
PAV	0.1259	0.1556	0.168	0.1721	0.185	0.1801	0.167

## Data Availability

The raw data supporting the conclusions of this article will be made available by the authors on request.
